# Cochlear Development; New Tools and Approaches

**DOI:** 10.3389/fcell.2022.884240

**Published:** 2022-06-23

**Authors:** Matthew W. Kelley

**Affiliations:** Laboratory of Cochlear Development, National Institute on Deafness and Other Communication Disorders, National Institutes of Health, Bethesda, MD, United States

**Keywords:** hair cell, organ of corti (OC), single cell RNA sequencing, cell fate, hearing

## Abstract

The sensory epithelium of the mammalian cochlea, the organ of Corti, is comprised of at least seven unique cell types including two functionally distinct types of mechanosensory hair cells. All of the cell types within the organ of Corti are believed to develop from a population of precursor cells referred to as prosensory cells. Results from previous studies have begun to identify the developmental processes, lineage restrictions and signaling networks that mediate the specification of many of these cell types, however, the small size of the organ and the limited number of each cell type has hampered progress. Recent technical advances, in particular relating to the ability to capture and characterize gene expression at the single cell level, have opened new avenues for understanding cellular specification in the organ of Corti. This review will cover our current understanding of cellular specification in the cochlea, discuss the most commonly used methods for single cell RNA sequencing and describe how results from a recent study using single cell sequencing provided new insights regarding cellular specification.

## Introduction

**FIGURE 1:**
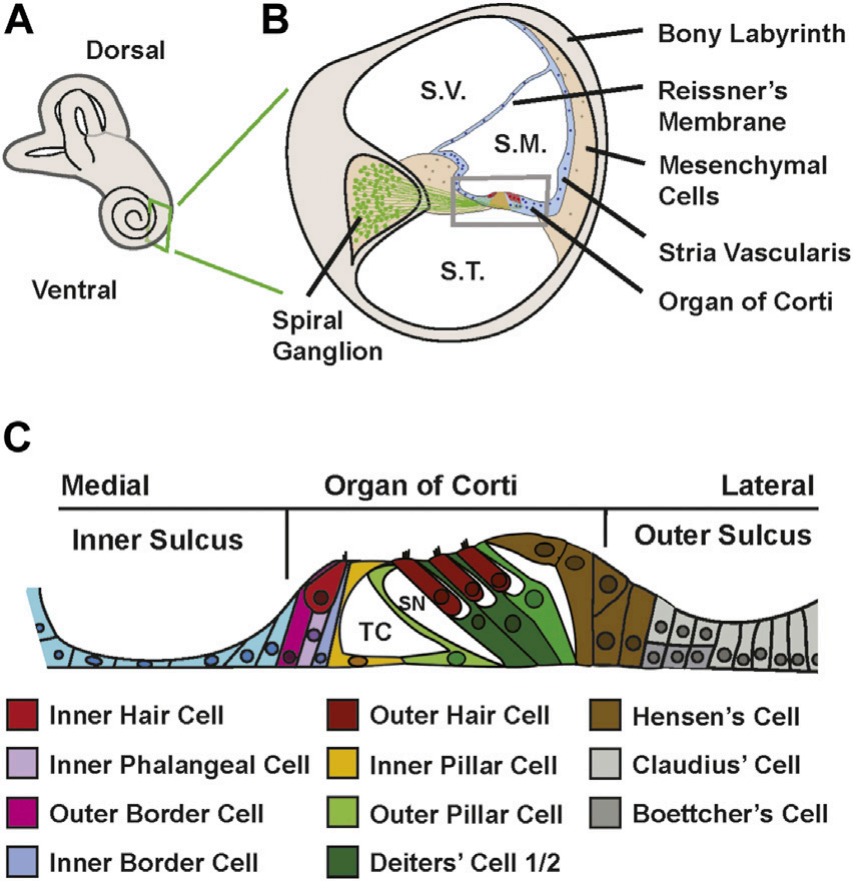
Structure of the cochlea and organ of Corti. **(A)** Diagram of the mammalian inner ear. The coiled cochlea is located on the ventral side. **(B)** Schematic cross-section of the mature cochlea. Sound waves travel up the cochlear spiral through the scala vestibuli (S.V.), pass through the scala media (S.M.) which contains the organ of Corti, and then travel back down the spiral through the scala tympani (S.T.). The scala media is a triangular-shaped structure with three morphologically distinct arms. Reissner’s membrane is a bilayered structure that separates the scala media from the scala vestibuli. The stria vascularis, which plays a key role in maintaining ionic homeostasis in the scala media, forms the majority of the lateral wall. The floor of the scala media contains the organ of Corti along with the adjacent inner and outer sulci (boxed area). HCs within the organ of Corti are innervated by neurons located in the spiral ganglion. The entire structure is encased in a capsule, the bony labyrinth. **(C)** Schematic of the floor of the scala media. Medial and lateral sides and the three divisions of the floor are indicated. The medial third of the floor is comprised of the inner sulcus, a monolayer of epithelial cells that represents the remnants of Kolliker’s organ. A similar structure, the outer sulcus comprises the lateral third. In the center of the floor is the organ of Corti. The medial edge of the organ contains a single IHC surrounded by inner phalangeal cells and inner and outer border cells. Moving laterally, single inner and outer pillar cells combine to form the tunnel of Corti (TC), a fluid filled space that is presumed to contribute to structural stability. In the lateral half of the organ, three OHCs are surrounded by Deiters’ cells which can be divided into 1^st^/2^nd^ row and 3^rd^ row cells at P1 based on gene expression. The Deiters’ cells create the spaces of Neul (SN) between the outer hair cells. . At the extreme lateral edge of the organ are Hensen’s cells which probably provide structural stability to the organ.

In mammals, sounds are initially perceived in the cochlea, the coiled structure that makes up the ventral portion of the inner ear (Figure 1A). . Structurally, the cochlear spiral contains three fluid filled chambers, the scala vestibuli, scala media and scala tympani which extend along it is long axis (Figure 1B). Located in the middle third of the floor of the scala media is the auditory sensory epithelium, also called the organ of Corti (OC). The OC is comprised of a band of cells that extends along the full length of the spiral (Figure 1C). Medial to the OC is the inner sulcus while the lateral third of the scala media floor contains the outer sulcus. In contrast with hair cell (HC) sensory epithelia in other vertebrates, and even in other parts of the mammalian inner ear, the OC is comprised of a highly rigorous mosaic of HCs and associated supporting cells (SCs) arranged in precise rows (Kelley, 2006; Groves and Fekete, 2012; Driver and Kelley, 2020). Moreover, both the HC and SC populations have become diversified to create two functionally distinct types of HCs and at least five distinct types of SCs (Figure 1C). Morphologically, the OC can be divided into two domains, a medial domain which is made up of inner hair cells (IHC) and surrounding inner phalangeal cells and border cells and a lateral domain typically containing three rows of outer hair cells (OHC), single rows of inner and outer pillar cells and three rows of Deiters’ cells. Adjacent to the third row of Deiters’ cells are the Hensen’s cells (Figure 1C). Whether these cells should be considered part of the lateral compartment of the OC or part of the outer sulcus is unclear (Chrysostomou et al., 2020). The medial domain appears similar in overall structure and function to other HC sensory epithelia. IHCs are separated from each other by interdigitating inner phalangeal cells while the medial and lateral sides of each IHC are contacted by border cells (Munnamalai and Fekete, 2020). Both inner phalangeal cells and border cells contact IHCs along their entire basolateral surface. Functionally, IHCs act similarly to afferent neurons releasing neurotransmitter in response to mechanical stimulation (Forge and Wright, 2002). In contrast, the function of the lateral domain has been modified to act as an active amplifier of incoming stimuli (Soons et al., 2015). OHCs are electrically motile, enhancing the stimulating signal that acts to activate adjacent IHCs (Hudspeth, 2008; Santos-Sacchi, 2019). The surrounding pillar cells and Deiters’ cells provide structural stability and intracellular spaces that are thought to be required for appropriate electromotility (Motallebzadeh et al., 2018; Ashmore, 2019; Santos-Sacchi, 2019). The emergence of OHCs, pillar cells and Deiters’ cells correlates with the evolution of high frequency hearing, leading to the general assumption that the structural changes in the lateral part of the OC are required for the perception of high frequencies and increased sensitivity (Okoruwa et al., 2008; Jahan et al., 2018).

As outlined above, by comparison with the elongated hearing organs in birds and some reptiles, the density of HCs along the medial-to-lateral axis of the auditory organ is decreased, a trend that also correlates with increased sensitivity and frequency range (Koppl and Manley, 2019). These changes suggest that the overall size of the sensory epithelium within the mammalian cochlear duct has decreased with evolution, a process that is likely to occur during the early development of the duct. The specific benefits of a single row of mechanosensors and three or four rows of amplifiers is not fully understood, but the exceptional performance of the mammalian cochlea is evident with champion hearing species like bats and toothed whales able to perceive sounds in excess of 150,000 Hz (Bohn et al., 2006; Wang et al., 2021). Finally, an unfortunate consequence of the evolution of the OC seems to be a complete loss of regenerative ability (Brignull et al., 2009; Denans et al., 2019). HC epithelia in all other vertebrate classes are able to robustly regenerate both HCs and auditory or vestibular function (Burns and Corwin, 2013; Burns et al., 2013) and even mammalian vestibular epithelia have been shown to be capable of a limited regenerative response (Forge et al., 1993; Warchol et al., 1993; Forge et al., 1998; Golub et al., 2012). In contrast, in the OC, all ability to regenerate HCs is lost prior to the onset of hearing (Kelley et al., 1995; Shi et al., 2013; Bramhall et al., 2014; Cox et al., 2014; Li et al., 2015; Maass et al., 2015; Tao et al., 2021). Whether this change arose as a result of the evolution of the OC’s diversity of cell types or precise patterning remains unclear.

Considering the diverse number of unique cell types that are required for the normal function of the OC, a better understanding of the pathways and processes that specify each type would provide valuable insights regarding cochlear development and possible strategies for regeneration. Unfortunately, because of its small size, the OC contains limited numbers of any particular cell type. A single mouse cochlea contains only approximately 1,000 IHCs while a human cochlea contains only 3 times more (Burda et al., 1988; Lim and Brichta, 2016). This limitation has significantly hindered efforts to understand the full diversity of cell types within the OC and, as important, the factors that direct cells along specific cellular lineages/fates. However, within the last 10 years, technical improvements have significantly increased the sensitivity of assays related to the capture and quantification of cellular genomic, transcriptomic and proteomic data (Cuevas-Diaz Duran et al., 2017; Butler et al., 2018; Kashima et al., 2020; Armand et al., 2021; Asada et al., 2021). As a result, it is now possible to profile development at the level of single cells. For organ systems such as the inner ear, these advances provide exciting new opportunities to significantly advance our understanding of the diversity of cells present within a specific organ and to examine how those cells are specified. This review will discuss our current understanding of the factors that specify cellular identity during development and then provide an overview of some of the most frequently used techniques for single cell transcriptional profiling. Finally, recent findings using single cell approaches to examine cellular diversity in the cochlea will be covered.

## Cochlear Development

**FIGURE 2:**
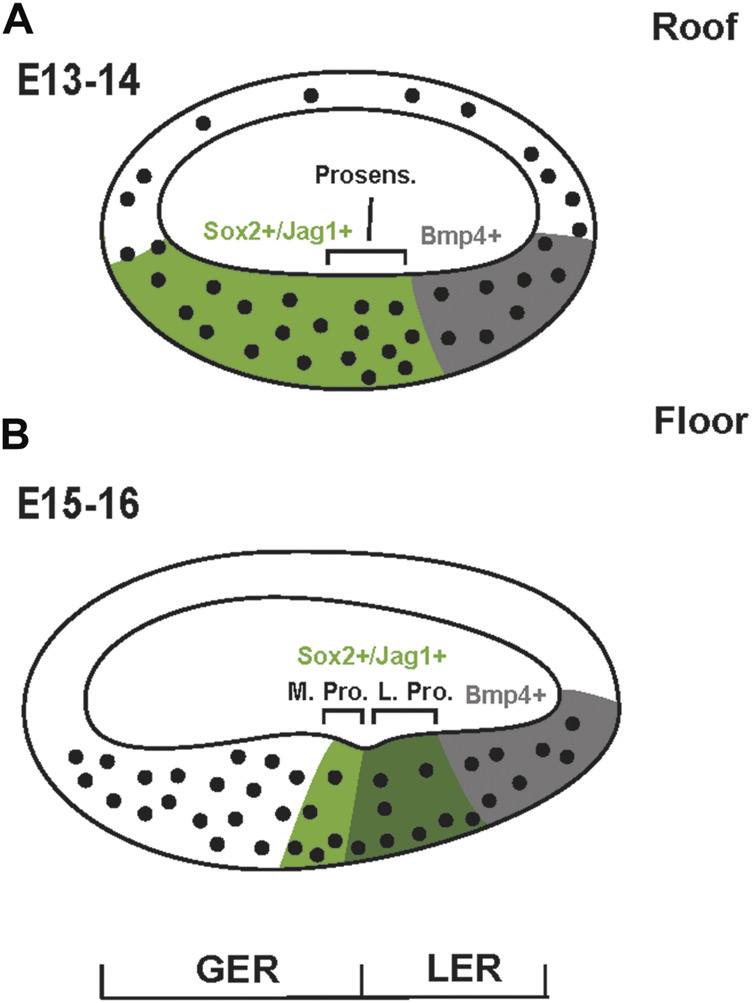
Development of the cochlear duct. **(A)** Schematic cross-section of the mouse cochlear duct on E13 or E14. While the entire duct is comprised of morphologically undifferentiated epithelial cells, the floor is already significantly thicker than the roof. Previous results have demonstrated that the floor can already be divided into Sox2^+^/Jag1^+^ and *Bmp4* ^*+*^ domains. The prosensory region is located at the lateral edge of the Sox2^+^/Jag1^+^ domain. **(B)** At E15-E16 the floor of the duct has become more differentiated. A physical notch in the epithelium defines greater and lesser epithelial ridges (GER and LER) which separate the Medial and Lateral Prosensory domains (M. Pro. and L. Pro.). By this stage, expression of Sox2 and Jag1 is largely confined to the prosensory domain. The lateral domain of *Bmp4*-expression still persists.

The inner ear is derived from the otocyst which begins as a placode located adjacent to the developing hindbrain (Wu and Kelley, 2012; Basch et al., 2016a; Driver and Kelley, 2020). Beginning around embryonic day (E) 10.5 in the mouse, the developing inner ear begins to extend a ducted protrusion from its ventral side. This duct then coils as it extends ultimately giving rise to the snail-shaped cochlea (Morsli et al., 1998). By E13 the duct is ovoid in cross-section and lined with morphologically undifferentiated pseudostratified epithelial cells (Lim and Anniko, 1985) (Figure 2A). However, the duct is not homogeneous. The dorsal surface, generally referred to as the floor, is noticeably thicker than the corresponding roof and will ultimately give rise to the OC and the flanking inner and outer sulci while the roof will develop as Reissner’s membrane and the stria vascularis (Figure 1A). While the cells of the duct appear largely homogeneous, molecular studies have demonstrated that at least two distinct populations of cells exist within the floor. A large population, marked by expression of the transcription factor Sox2 and the Notch ligand Jagged1 (Jag1), extends roughly two thirds of the distance from the medial edge while a smaller Bmp4^+^ population covers the final third of the medial-to-lateral axis (Morrison et al., 1999; Kiernan et al., 2005b; Brooker et al., 2006; Kiernan et al., 2006; Chang et al., 2008; Dabdoub et al., 2008; Ohyama et al., 2010). Based on persistent expression of Bmp4, the lateral-most cells are believed to give rise to the cells of the outer sulcus (Figure 1). The cells that will give rise to the OC, referred to as prosensory cells because of their unique ability to give rise to HCs and SCs (Kelley et al., 1993), form near the middle of the duct in the lateral half of the Sox2/Jag1^+^ population. As development proceeds, several changes occur nearly simultaneously within the Sox2/Jag1^+^-population. First, expression of Sox2/Jag1 becomes restricted to the central region of the duct, correlating with the prosensory domain (Morrison et al., 1999; Kiernan et al., 2001; Dabdoub et al., 2008). Second, in a gradient that begins at the apex of the cochlear duct around E12.5, cells within the prosensory domain upregulate expression of the cell cycle inhibitor, Cdkn1b (formerly p27^kip1^), leading to cell cycle exit and the formation of the zone of non-proliferation (ZNP) that reaches the base of the cochlea by E14.5 (Ruben, 1967; Chen and Segil, 1999; Lee et al., 2006). Concomitant with the arrival of the wave of cell cycle exit at the base, the prosensory region separates into medial and lateral domains (Figure 2B) (Pirvola et al., 2002; Bermingham-McDonogh et al., 2006; Huh et al., 2012; Yang et al., 2019). The specific fates of cells within each domain are consistent with the evolutionary changes that have occurred within the mammalian lineage. Fate mapping of the lateral prosensory domain, which expresses both Fibroblast Growth Factor Receptor 3 (Fgfr3) and Prospero Homolog 1 (Prox1), indicates that these cells will give rise to OHCs, pillar cells and Deiters’ cells (Kolla et al., 2020). While cells within the medial domain, are believed to give rise to IHCs, inner phalangeal cells and border cells.

The factors that specify the medial and lateral domains are unknown but may be linked to the overall specification of cellular identities along the medial-to-lateral axis of the cochlear duct. As discussed, cells located at the lateral edge of the duct express a known morphogen, Bmp4, while cells throughout the duct express the Bmp4 receptors *Alk3* and *Alk6* (Ohyama et al., 2010). Deletion of these receptors leads to changes in gene expression that are consistent with increased medial phenotypes and decreased lateral phenotypes. Consistent with these results, cochlear explants treated with the Bmp receptor antagonist dorsomorphin show a decrease in the size of the Prox1^+^-domain (lateral) and an apparent increase in the size of the medial domain (Munnamalai and Fekete, 2016). Similarly, treatment of cochlear explants with the Glycogen Synthase Kinase 3 (Gsk3) inhibitor CHIR99021 induces decreases in *Bmp4* expression and phosphorylation of the Bmp4 target, SMAD1, and an OC that contains multiple rows of IHCs at the expense of OHCs (Ellis et al., 2019). Fate mapping demonstrates that this phenotype arises, at least in part, as a result of a shift in the medial-lateral boundary within the OC, suggesting that the gradient of Bmp4 plays a key role in the establishment of the medial-lateral axis. Consistent with this hypothesis, addition of Bmp4 to CHIR99021-treated explants partially rescues the phenotype. Gsk3β has been shown to modulate a number of different signaling pathways, in particular it acts as a Wnt inhibitor, meaning that treatment with CHIR99021 can act as a Wnt agonist (Doble and Woodgett, 2003; Patel and Woodgett, 2017). In fact, several Wnts have been shown to be expressed in the medial half of the cochlear duct, suggesting that Wnt may act in the specification of medial identity, possibly in a counter-gradient to Bmp4 (Shi et al., 2014; Geng et al., 2016). Consistent with this idea, treatment of cochlear explants with CHIR99021 for 24 h beginning on the equivalent of E13.5 induces a significant increase in IHCs but no decrease in OHCs (Munnamalai and Fekete, 2016). This phenotype differs from the experiments described above in terms of time of exposure and dosage but are consistent with Wnts playing a role in specification of the medial domain that is independent of the down-regulation of Bmp4 at the lateral edge of the duct. However, results from other studies suggest that the increased representation of medial OC cells following CHIR99021 treatment may not be mediated through Wnt signaling. First, application of a Wnt agonist targeting TCF transcriptional activation directly, did not replicate the phenotype observed following treatment with CHIR99021 (Ellis et al., 2019). Perhaps more compelling, while genetic deletion of *β-catenin* (a key regulator of Wnt function and the target of Gsk3β) induced a shift in the OC medial-lateral boundary, the shift was in the same direction, medially, as was observed following CHIR99021 treatment (Jansson et al., 2019). Moreover, the same study provided evidence that the phenotype is dependent on β-catenin’s role in cell adhesion, rather than as an activator of Wnt signaling. So, to summarize, these results are consistent with a role for Bmp4, likely acting as a morphogen, in the specification of cellular identities along the medial-lateral axis of the cochlea. While Wnts clearly also play a role in cochlear development and patterning, whether this includes acting as a counter gradient to Bmp4 remains to be determined.

An additional signaling pathway that may play a role in medial-lateral patterning is the Notch pathway, and in particular, the Notch ligand Jag1. Notch signaling plays multiple roles in inner ear development (Kiernan, 2013; Brown and Groves, 2020; Daudet and Zak, 2020). Inductive interactions between Jag1 and Notch1 during early stages of otocyst development specify vestibular and auditory prosensory patches (Brooker et al., 2006; Kiernan et al., 2006). Also, following the onset of their differentiation, HCs upregulate expression of two Notch ligands, *Delta-like 1* (*Dll1*) and *Jagged2* (*Jag2*) which bind to and activate Notch1 in adjacent prosensory cells, inhibiting those cells from forming as HCs and forcing them towards a SC fate in a classic example of the role of Notch in mediating lateral Inhibition (Lanford et al., 1999; Kiernan et al., 2005a). However, the role of Jag1 during cochlear development is more complicated. As the OC develops, Jag1 expression is down-regulated in HCs but maintained in SCs (Lewis et al., 1998; Morrison et al., 1999), a pattern that is inconsistent with a role in lateral Inhibition. Consistent with this observation are the results of studies that have examined cochlear phenotypes in mice with ENU-induced point mutations in *Jag1*. *Slalom, Headturner* and *Ozzy* mice all carry point mutations in the extracellular domain of *Jag1* leading to missense mutations but none of these mice have cochlear phenotypes that suggest defects in lateral inhibition (Kiernan et al., 2001; Tsai et al., 2001; Vrijens et al., 2006). Instead, all three lines show a consistent phenotype of increased IHCs and decreased OHCs. In addition, atypical HCs, often located in the pillar cell region are also observed. These results are consistent with changes in patterning along the medial-lateral axis of the OC. However, considering that Jag1 is expressed in all SCs during cochlear development, a mechanism for its role in patterning remains unclear. Finally, conditional deletion of *Jag1* in the cochlea beginning on E14.5 has no obvious effect on the patterning of HCs and SCs or on the ratio of IHCs to OHCs (Chrysostomou et al., 2020). Since the medial-lateral axis is thought to be specified prior to E14.5 (Basch et al., 2016b; Ohyama et al., 2010), this result is not inconsistent with a role for Jag1 in axial patterning, but it also, unfortunately, doesn’t provide any additional insights regarding the role of Notch in this process.

Terminal mitosis of the prosensory cells and specification of the medial-lateral axis is followed by cellular differentiation which begins around E14.5. But in contrast with the gradient of cell cycle exit, which extends from apex-to-base, differentiation extends in a gradient from base-to-apex (Rubel, 1978). As a result, prosensory cells located at the apex of the cochlea remain in a post-mitotic, undifferentiated state for several days in mouse and probably longer in humans (Chen et al., 2002). The biological benefits, if any, for this unconventional pattern of terminal mitosis and differentiation are unclear. While the factors the regulate the timing and pattern of cellular differentiation remain poorly understood, recent studies have demonstrated important roles for *Sonic hedgehog* (*Shh*), and two sets of interacting signaling molecules, *let-7/Lin28b* and *Activin A* (*Inhba*)/*Follistatin* (*Fst*) (Bok et al., 2013; Golden et al., 2015; Prajapati-DiNubila et al., 2019) in this process. Prior to E14, the developing spiral ganglion (Figure 1A) acts as a source of Shh which blocks HC differentiation in the adjacent cochlear epithelium. *Shh* is then down-regulated in a basal-to-apical gradient the parallels differentiation (Liu et al., 2010; Bok et al., 2013). Modulation of Shh signaling either *in vivo* or *in vitro* alters the timing and patterning of HC formation and also leads to hearing deficits (Driver et al., 2008). Interestingly, the basal-to-apical pattern of cellular differentiation is flipped in Shh conditional mutants, resulting in a gradient that more closely parallels the gradient of terminal mitosis (Bok et al., 2013).

In contrast with Shh, which arises outside the epithelium, developing prosensory cells express *let-7/Lin28b* and *Inhba/Fst*. *Lin28b* and *Fst*, both of which inhibit HC differentiation, are broadly expressed throughout the cochlear duct prior to E14 (Golden et al., 2015; Prajapati-DiNubila et al., 2019). Concomitant with the onset of differentiation, expression of *let-7*, a microRNA which targets cell cycle genes in addition to *Lin28b*, and *Inhba*, which codes for a secreted antagonist of Follistatin, increases beginning in the base of the cochlea. Perturbation of either of these pathways leads to changes in the timing and patterning of HC differentiation and can also influence stemness in some SCs. Whether either of these pathways is controlled through Shh has not been determined however, evidence from the CNS suggests that Shh signaling may act to inhibit *let-7* expression (Tanno et al., 2016).

The first cells to differentiate are generally believed to be IHCs located near the base of the cochlea. Certainly, molecular markers for HCs first appear in the mid-basal IHC region (Montcouquiol and Kelley, 2003). HC differentiation then proceeds in gradients that extend primarily apically but also towards the base (Rubel, 1978). In addition, HCs differentiate in a medial-to-lateral gradient at any given position along the spiral. SCs appear to differentiate in the same pattern, although this has been harder to study, largely because SC differentiation is a longer process, extending, in the mouse, through the first two postnatal weeks (Lim and Anniko, 1985; Tucker et al., 1993; Chen et al., 2018), and because definitive markers of SC differentiation are limited. Instead, many of the genes that are expressed in SCs are also expressed in prosensory cells.

While IHCs are thought to be the first cells to differentiate, it is important to consider that inner pillar cells may actually be specified, at some level, prior to the onset of IHC development (Thelen et al., 2009). Early histological studies of cross-sections through the embryonic cochlear duct often noted a physical notch located in the general region of the future OC which creates two clusters of epithelial cells referred to as the greater (medial) and lesser (lateral) epithelial ridges (Figure 2B). The notch arises because the epithelium constricts from a multilayered pseudostratified epithelium down to a single cell. A study examining early development of the cochlear duct in rats provided strong evidence that this cell represents the future inner pillar cell. Moreover, by labeling for the presence of polysaccharides, which are strongly expressed in mature inner pillar cells, the authors demonstrated that this single cell contained polysaccharides before the differentiation of IHCs. So, while these cells must go through extensive developmental changes to assume the morphology of inner pillar cells, these results suggest that some level of cellular specification has occurred in pillar cells prior to the development of IHCs. However, these cells are not yet committed to an Inner pillar cell fate as Fgf8-mediated activation of Fgfr3 beginning around E15 is required to both prevent these cells from converting to a HC fate and to drive their subsequent development (Mueller et al., 2002; Jacques et al., 2007; Mansour et al., 2009; Mansour et al., 2013).

As development continues, cell-cell interactions between HCs and surrounding prosensory cells act to regulate the subsequent development and patterning of the OC. First, as discussed, HCs express Dll1 and Jag2, which activate the Notch pathway in surrounding cells to inhibit those cells from developing as HCs (Lanford et al., 1999; Kiernan et al., 2005a; Brooker et al., 2006). In addition, HCs generate largely unknown inductive signals that recruit surrounding cells to develop as SCs (Woods et al., 2004). Activation of Notch has been shown to play a role in this inductive process (Campbell et al., 2016), and, as mentioned in the previous paragraph, in the case of the developing PCs and DCs, Fgf8 secreted from IHCs is also required for normal formation.

Following the initial onset of differentiation, both HCs and surrounding SCs go through a protracted period of differentiation that, in the mouse, lasts through the first and second postnatal weeks. Over the course of this process, HCs will develop mature stereociliary bundles, mechanotransductive channels and synaptic structures while SCs will undergo significant morphological changes leading to the formation of the tunnel of Corti and the opening of the spaces of Neul (Inoshita et al., 2008). While this process appears to occur in a similar fashion along the entire length of the cochlear spiral, subtle differences related to frequency tuning are evident in the size and morphology of both HCs and SCs (Vater and Kossl, 2011; Yarin et al., 2014). Therefore, in terms of the genetic regulation of cochlear development, it seems reasonable to expect that both conserved and variable genetic signaling pathways must be activated in spatially specific patterns to drive the formation of the mature structure. The observation that some aspects of OC structure and function vary along the tonotopic axis emphasizes the need to be able to study cochlear development at the single cell level.

## Single Cell Analyses–A New Approach to Answer Old Questions

The feasibility of profiling mRNA expression at the level of single cells was initially demonstrated by Eberwine and others (Eberwine et al., 1992) who dissociated adult hippocampal neurons and then performed patch clamp recordings. At the end of each recording, cytoplasm from the cell was drawn into the patch pipette which contained a mixture of primers, nucleotides and reverse-transcriptase. Following multiple rounds of amplification, the resulting cDNA was used to screen for expression of known neuronal genes. While this study demonstrated that isolation and detection of mRNAs from single cells was possible, the approach was laborious and inefficient. In the 30 years since then, multiple advances, including the development of Illumina sequencing, the sequencing of the mouse and human genomes, and increased efficiency of generation and subsequent amplification of cDNA has improved and simplified the ability to profile mRNA expression from single cells.

A key first step in any effort to generate profiles at a single cell level is a determination of whether individual cells need to be isolated, and if so, what is the best method. Laser-capture microdissection, which offers the advantage of providing spatial information about the collected sample, uses laser light to excise a specific region(s) from a tissue section (Cheng et al., 2013; Datta et al., 2015; Bhamidipati et al., 2022). The excised region is then captured and digested to collect mRNAs. However, the fixation process often leads to degradation of mRNA leading to low efficiency. Moreover, depending on the thickness of the section and the plane of the cut, it is possible to collect mRNA from two distinct cells in a single section. More frequently, tissues are dissociated to generate isolated single cells. The ease with which this can be accomplished varies depending on the nature of the tissue and its age. This approach generally leads to high mRNA yields and reasonable efficiency. However, these benefits come at the loss of positional information and in some circumstances an induction of stress responses which can skew the resulting transcriptomic data (Machado et al., 2021). Finally, for some tissues, such as the mature brain, the intertwining of cells and the existence of long axonal projections may make dissociation extremely difficult leading to cell damage, stress and possibly apoptosis. For these types of tissues, rather than isolating single cells, it can be more expeditious and provide higher purity to isolate single nuclei (Habib et al., 2016; Ding et al., 2020). Moreover, since one of the first steps in isolating single nuclei is cell lysis, stress responses are not initiated. However, since nuclei contain lower amounts of mRNA, results from single nuclei typically have less information in terms of gene expression and may not accurately reflect the full spectrum of mRNAs expressed in the cell. Finally, the technique pioneered by Eberwine et al., now called Patchseq, can still be applied although this approach remains labor intensive and can have low efficiency (Cadwell et al., 2016; Fuzik et al., 2016). Each of these approaches has advantages and disadvantages that often must be weighed against the nature of the tissue to be profiled as well as the specific questions to be addressed. In particular, in tissues such as the inner ear, where cellular structure and function are often directly related to position, the loss of positional information may need to be addressed. However, while beyond the scope of this review, novel methods are or will soon be available to generate comprehensive transcriptomic data while maintaining positional identity (Rodriques et al., 2019; Dar et al., 2021; Lohoff et al., 2022).

Concomitant with the consideration of how to isolate cells are the questions of how to collect those cells and what type of expression data is needed. Options for collections of cells includes physical picking of cells using pulled micropipettes, serial dilution of cells into plates containing 96, 384 or thousands of individual wells, fluorescent activated cell sorting (FACS), or microfluidic approaches that can capture single cells in individual wells or aqueous droplets. In general, there is an inverse relationship between the number of cells captured and the number of genes detected and number of sequencing reads per gene.

A final consideration is the depth of data that will be needed for a specific project. For instance, to characterize different cell types from a tissue comprised of disparate cellular phenotypes, quantifying a percentage of the genes that are expressed in each cell may be sufficient. But to explore differences in splice variants or isoform usage in a population of phenotypically similar cells, significantly deeper sequencing may be required. As will be discussed below, the approach used to amplify and label mRNAs from each cell can be customized at different points based on the specific question being asked.

Currently the two most common methods for capturing mRNAs from single cells are SMARTseq (currently on version 4) and bead-based bar coding, which also uses SMARTseq technology. Each will be discussed briefly below.

SMARTseq (Switching Mechanism at 5′ End of RNA Template) relies on the template switching (TS) activity of reverse transcriptases (RT) such as Maloney Murine Leukemia Virus (MMLV)-RT (Ramskold et al., 2012; Picelli et al., 2013; Picelli et al., 2014). Briefly, mRNAs are captured from lysed individual cells and reverse transcription is initiated using oligo-dT primers that also add a PCR primer sequence. When the RT reaches the 5′ end of each mRNA strand, several additional nucleotides, typically cytidines, are added to the cDNA strand. These extra bases hybridize with a TS-oligo containing another PCR primer and the RT then switches to the TS-oligo to form a cDNA that contains primer sites on each end. Because TS requires that the RT reach the 5’ end of each mRNA, SMARTseq generates full length cDNAs. These cDNAs are then amplified using polymerase chain reaction followed by library preparation, shearing and Illumina-sequencing. The resulting data is then aligned to current genomic builds. A distinct advantage of SMARTseq approaches is the generation of full-length data and considerably higher read depth and gene detection per cell.

Bead-based bar-coding approaches are generally used in combination with approaches that seek to collect significantly greater numbers of cells, primarily using aqueous droplets to capture cells (Klein et al., 2015; Macosko et al., 2015; Zheng et al., 2017). Briefly, isolated cells and oligo-coated gel beads-in-emulsion (GEMs) are flowed in a microfluidic apparatus designed to generate droplets that contain one bead and 1 cell. Each bead is arrayed with multiple oligos that all contain the same nucleotide bar code along with an oligo-dT region for capture of mRNAs. Thousands of cells can be collected in a single run. Once the run is complete, cells are lysed and cDNAs are generated, largely as described above except that the molecular bar code is incorporated at the 3′ end of the resulting cDNAs. Following library preparation, cDNAs are sheared prior to sequencing. Since bar codes are only present at the 3′ end of each cDNA, relatively short 90-base pair reads are used to quantify gene expression based solely on 3’ sequences. The presence of the barcode with each sequence allows every cDNA to be assigned to a particular cell. So, while no data on isoform usage or splice variants can be obtained, thousands of cells can be sequenced in a single run.

While the choice of which isolation and sequencing approaches to use should be made based on the biological question(s) being addressed, experience from our laboratory and others has provided some insights regarding cochlear cells. As is the case for many tissues, during embryonic development, both epithelial and mesenchymal cells are easily dissociated and, more importantly, quite robust in terms of their ability to survive following dissociation. In our experience, single cell suspensions of viable cells can be obtained from mouse cochlear cells aged P5 or less (Burns et al., 2015; Kolla et al., 2020). Therefore, at embryonic and early post-natal ages, the approach can be decided on, largely, based on the question that is being asked. For questions related to the identification of unique cell types or transcriptional profiles, a higher-volume, lower read depth approach, such as 10X captures could be used while questions related to discovery of novel mRNA transcripts or alternative splicing could also be addressed using lower-throughput approaches combined with SMART-Seq or PacBio long read sequencing.

At older ages, we have observed increasing difficulty in obtaining high numbers (> than 1,000 from a single experiment) of good quality single cells, in particular HCs. However, we have successfully isolated adult SCs following FACS enrichment (Burns et al., 2015; Hoa et al., 2020). Therefore, to obtain high numbers of both HCs and SCs from cochleae older than P5, a single nucleus approach would be preferable. Similar studies have demonstrated that this approach can be used to isolate cells from the stria vascularis (Korrapati et al., 2019; Gu et al., 2020; Taukulis et al., 2021). For lower throughput but greater depth, several laboratories have combined dissociation, visual identification and individual selection using micropipettes to collect cells of known phenotypes (Liu et al., 2014; Li et al., 2018; Ranum et al., 2019). While the numbers of cells that can be collected using this technique is limited, the potential for a more comprehensive survey of transcriptional expression along with novel alternative splicing and even epigenomic profiling (see below) is high.

Finally, as mentioned above, for many aspects of cochlear development and function, the spatial location of each cell fundamentally influences that cell’s phenotype and physiology. Unfortunately, the relatively small number of cells in a single cochlea limits the ability to collect cells from a sub-region (such as a specific frequency range). Broader isolations, such as basal or apical halves of the cochlea have been successfully executed providing data on difference in developmental progression. Novel approaches in which a matrix of GEM-type beads are adhered to a glass slide, are currently in development (Rodriques et al., 2019; Marshall et al., 2022; Wang et al., 2022; Zhao et al., 2022). For this approach, the bar-code for each bead is determined initially and then a frozen tissue section is placed on top of the matrix. mRNAs are captured as described for the 10X Genomics system and following sequencing individual mRNAs can be assigned to a specific position in the initial tissue section. Current bead sizes are approximately 50 μm, which prevents single cell resolution for cochlear cells, but technological advances may decrease bead size to 5 μm in the near future.

While RNA sequencing has been the focus of most single cell studies, it is now possible to also assess the epigenetic state of a single cell using Assay for Transposase Accessible Chromatin Sequencing (ATACSeq) alone or in combination with RNAseq (Macaulay et al., 2017; Mezger et al., 2018; Satpathy et al., 2019). In addition, the development of long-read technologies in which entire cDNAs are sequenced, such as Nanopore MinIon or Pacific Biosciences Sequel platforms, have the potential to provide data on novel splice variants at the level of single cells (Lu et al., 2016; Chu et al., 2017; Midha et al., 2019). Finally, expression of a significant number of proteins can be now be assayed at the single cell level using shotgun mass spectrometry (Zhu et al., 2019).

## Cellular Diversity in the Cochlear Duct; Improved Resolution Using Single Cell RNAseq

**FIGURE 3:**
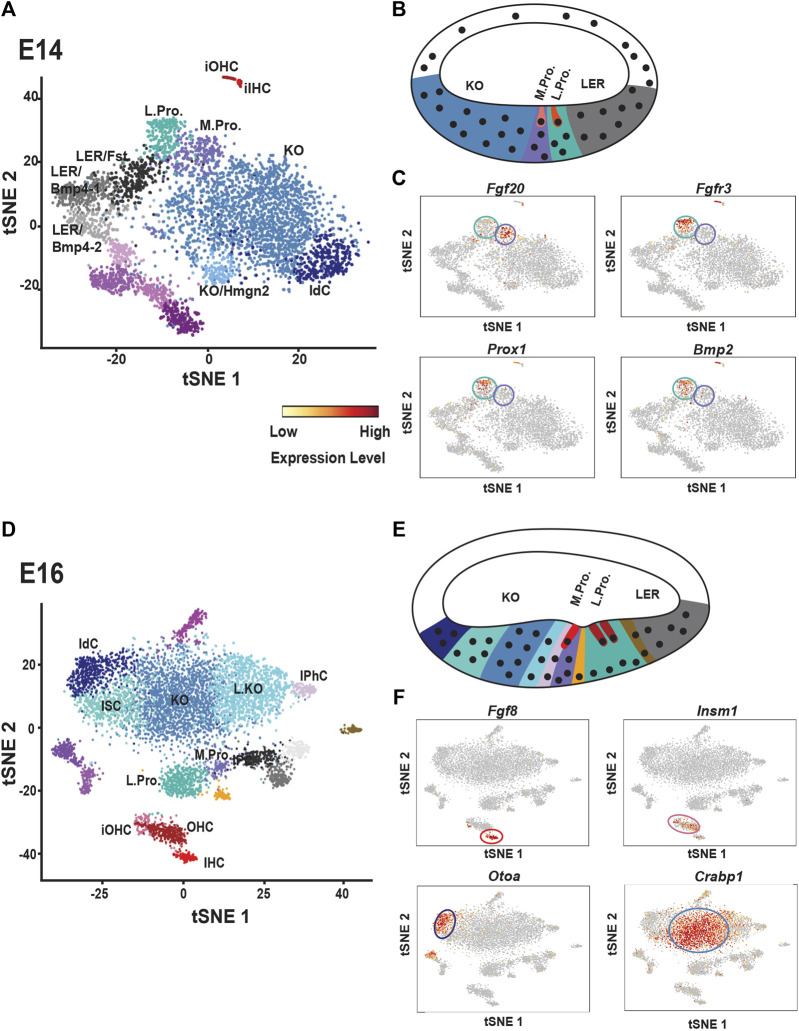
Single cell analysis of cochlear development at E14 and E16. **(A)** t-distributed stochastic neighbor embedding (tSNE) plot of cochlear single cells collected at E14. Transcriptionally unique clusters are indicated in different colors. Clusters with transcriptional similarity, such as those determined to be located in Kolliker’s organ (KO) are colored similarly. Clusters discussed in the text are labeled. See text for details. **(B)** Schematic cross-section through the base of the cochlear duct at E14. The general locations of Kolliker’s organ (KO), the medial and lateral prosensory cells (M. Pro. and L. Pro.) and the lesser epithelial ridge cells (LER) which will develop as the outer sulcus, are indicated and color-coded to match cell clusters in **(A)**. Note: KO and M.Pro. together constitute the GER while LER technically includes L.Pro. as well as the region labeled LER. **(C)** Feature plots showing expression of medial (*Fgf20*) and lateral (*Fgfr3, Prox1, Bmp2*) prosensory markers in the single cell data. Medial prosensory cells are circled in purple and lateral prosensory cells are circled in Aqua. Compare each feature plot with the plot in **(A)** Scale bar indicates level of mRNA expression per cell. Abbreviations: iOHC: immature outer hair cells, OHC: outer hair cells, IHC: inner hair cells, IdC: interdental cells. **(D)** tSNE plot of cochlear single cells collected at E16. As discussed in the text, note the increased diversity of cell types within KO and the transcriptional similarity between Inner Phalangeal Cells (IPhC) and KO cells. **(E)** Schematic cross-section through the cochlear duct at E16. Immature IHCs and OHCs (shades of red) can be identified as can developing inner pillar cells (gold) and some inner phalangeal cells (pink). However, medial prosensory cells (purple) are still present as well. Cells in KO have become transcriptionally heterogenous with different cell clusters located along the medial-to-lateral axis of the duct. See text for details. **(F)** Feature plots showing expression of makers for developing IHCs (*Fgf8*) and OHCs (*Insm1*) as well as two genes, *Otoa* and *Crabp1* that are restricted to specific regions of KO cells. Abbreviations: IPC: inner pillar cells, ISC: inner sulcus cells All tSNE and feature plot images were generated using gEAR (umd.gear).

As discussed above, the cochlear duct goes through multiple rounds of cellular specification during development. Based on gene expression studies, at E14 the floor of the duct can divided into three regions from medial-to-lateral: Kolliker’s organ, the prosensory domain and the Bmp4+ lesser epithelial ridge (LER) (Ohyama et al., 2010). However, a recent single cell analysis of cell types within the duct at E14 indicated a greater degree of cellular diversity (Kolla et al., 2020). Kolliker’s organ was fairly homogenous at this time although two small additional clusters were observed (Figure 3A). One of the smaller clusters represented immature interdental cells which have already begun to generate components of the tectorial membrane and the other cluster expressed the high mobility group nucleosome-binding chromosomal protein *Hmgn2*, suggesting possible changes in transcriptional activity within these cells. Similarly, the *Bmp4* ^*+*^ LER resolved into three different clusters. All three expressed *Bmp4*, as well as the Bmp-related molecule *Follistatin* (*Fst*), but were transcriptionally distinct based on expression of other genes. The potential different roles and/or fates of these distinct groups remain to be determined.

Finally, as discussed above, the prosensory domain resolved into two separate clusters (Figures 2A, 3C). Consistent with previous studies, *Fgfr1, Fgfr3, Prox1, Bmp2, Ngfr *and *Nrcam* were all restricted to what was classified as the lateral prosensory domain (von Bartheld et al., 1991; Mueller et al., 2002; Pirvola et al., 2002; Bermingham-McDonogh et al., 2006; Hwang et al., 2010; Harley et al., 2018) (Figure 3C). In contrast, the medial domain expressed fewer unique transcripts with *Fgf20* the most differentially expressed gene in this domain although previous studies indicated the *Fgf20* is expressed in the lateral domain as well prior to E14 (Hayashi et al., 2008; Huh et al., 2012).

By 2 days later, at E16, additional OC cell types can be identified (Figure 2D). Along with IHCs and OHCs, a specific population of inner pillar cells separates from other lateral prosensory cells. Deiters’ cells have not differentiated sufficiently to separate from lateral prosensory cells and a population of medial prosensory cells is still present as well. However, a population of inner phalangeal cells, that clusters independently from the medial prosensory cells, is also present. Interestingly, this cluster of cells shows a greater degree of transcriptional similarity with cells in Kolliker’s organ by comparison with medial prosensory cells. The most reasonable explanation for this result is that differentiated inner phalangeal cells become transcriptionally similar to cells in Kolliker’s organ, in particular cells located at the lateral edge of Kolliker’s organ (see below), but an alternative possibility is that some inner phalangeal cells are derived from Kolliker’s organ rather than medial prosensory cells. Fate-mapping using *Fgf20* ^*cre*^ mice, a prosensory marker, indicates that most inner phalangeal cells are derived from prosensory cells (Landin Malt et al., 2019) but functional data has demonstrated that cells from Kolliker’s organ can develop as inner phalangeal cells following ablation of the native inner phalangeal cell population (Mellado Lagarde et al., 2014).

**FIGURE 4:**
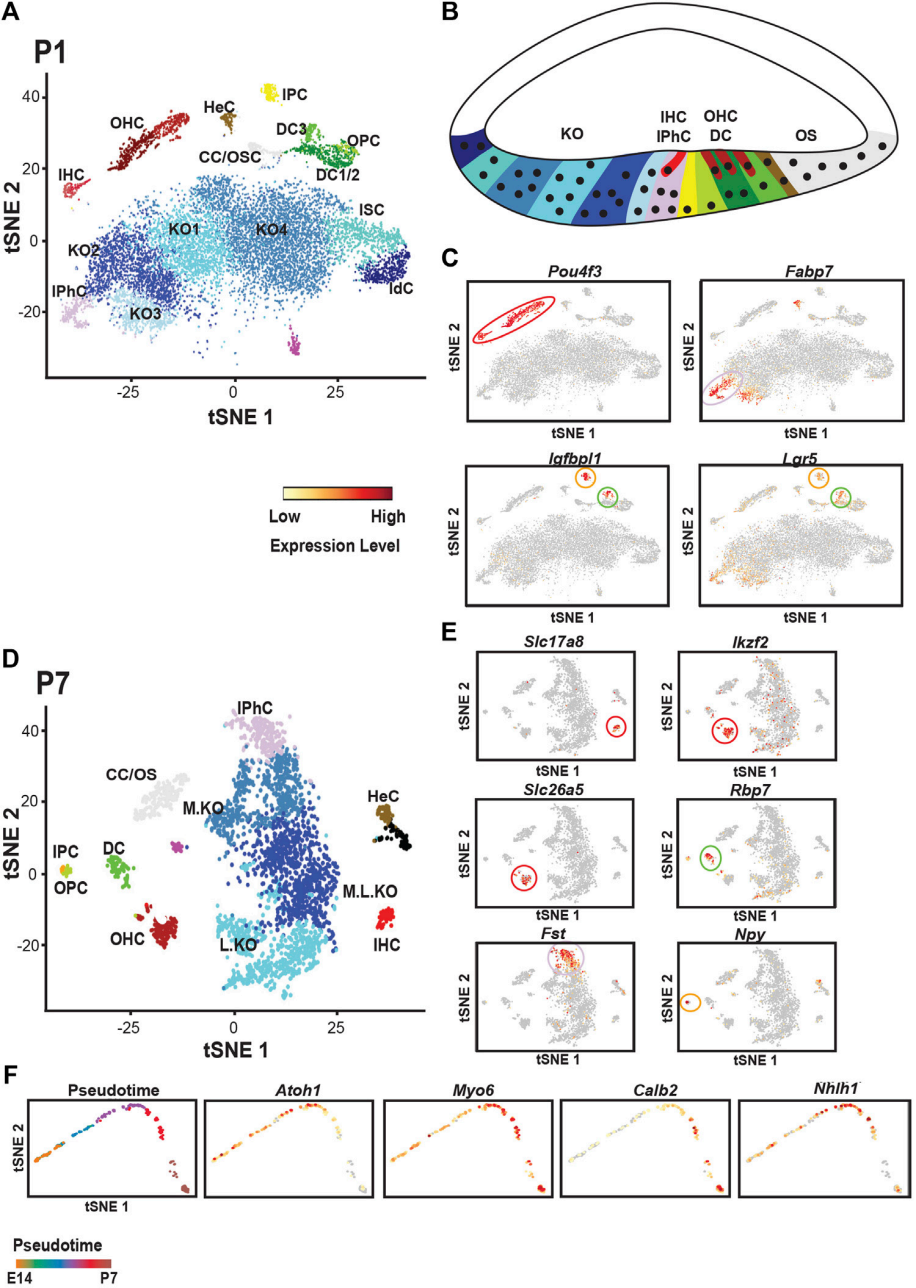
Single cell analysis of cochlear development at P1 and P7. **(A)** tSNE plot for single cells collected from the floor of the cochlear duct at P1. All of the known cell types within the organ of Corti can be identified as can Hensen’s and Claudius’ cells (HeC and CC). In addition, multiple transcriptionally distinct cell types are identified in Kolliker’s organ (KO1-4), Interdental cells (IdC) and Inner Sulcus cells (ISC)). **(B)** Schmatic of the cochlear duct at P1 with positions of cells from A indicated by color. The basic pattern within the organ of Corti is present although many of the supporting cells have not fully differentiated at this time point. **(C)** Feature plots illustrating expression of marker genes for hair cells (*Pou4f3*), inner phalangeal cells (*Fabp7*), and inner pillar cells/Deiters’ cells (*Igfbp1* and *Lgr5*). **(D)** tSNE plot for cochlear single cells collected at P7. All of the cell types within the organ of Corti can be identified. Inner and outer pillar cells (IPC, OPC) are very similar transcriptionally, a change from P1. In addition, all three rows of Deiters’ cells cluster together suggesting that the transcriptional differences between Deiters’ cells in rows one and two versus three at P1 may be a result of differences in the timing of their development. In contrast with P1, the number of unique cell clusters in Kolliker’s organ has decreased to three. For relative positions of different cells types in the cochlea, refer to Figure 1B. **(E)** Feature plots illustrating marker genes for different cell types in the organ of Corti at P7. *Slc17a8* marks IHCs while OHCs express *Ikzf2* and *Slc2615* (*Prestin*). Deiters’ cells uniquely expressed the retinol binding protein *Rbp7*, inner phalangeal cells express *Fst* and inner pillar cells express *Npy*. **(F)** Pseudotime projection for developing OHCs collected from all four time points (E14, E16, P1 and P7). Color coding in upper left panel indicates relative age across the pseudotime projection from E14 to P7. All other panels show expression of specific genes across pseudotime (see scale bar in 4C). *Atoh1* is down-regulated as hair cells develop, *Myo6* and *Calb2* are up-regulated as hair cells mature and the transcription factor *Nhlh1* shows transient expression. All tSNE and feature plot images were generated using gEAR (umd.gear).

Finally, consistent with ongoing development of the cochlea, single cell analysis at P1 indicated no prosensory cells. But all of the major OC cell types, inner phalangeal cells, both types of pillar cells, Deiters’ cells (in two clusters, Deiters’ cell1/2 and 3), Claudius and Hensen’s along with IHCs and OHCs are present (Figure 4A). A further analysis at P7 clustered all Deiters’ cells together suggesting a convergence in transcriptional profiles as Detiers’ cells mature (Kolla et al., 2020). However, because of the difficulty in collecting cells at P7, the overall size of the data set was smaller by comparison with early ages which may have obscured transcriptional differences between different Deiters’ cell types. Overall, the results of the study by Kolla et al. (2020) provided novel in depth transcriptional profiles for each of the known cell types within the OC including new markers for several cell types. However, with the exception of the separation between Deiters’ cells located in rows one and two versus row three at P1, which was already known, novel cell subtypes were not described. While this may indicate limited heterogeneity within these cell populations, several caveats should be considered. First, the numbers of cells collected for each cell type was relatively low. As will be discussed below, a much greater number of cells were collected from Kolliker’s organ which revealed multiple subtypes. In addition, since all the cells from each time point were analyzed together, subtle differences in transcriptional expression could have been obscured. Additional analyses in which cells within a single cluster are isolated and reanalyzed could reveal individual differences that were masked in the analysis of the larger data set. Along the same lines, the addition of more OC cells could improve cellular resolution. Finally, as discussed, sequencing data derived using the 10x Genomics approach is limited to the 3′ end of each transcript. So, differences in splice variant or isoform usage cannot be determined. Therefore, additional studies using long-read platforms could be applied to reveal additional heterogeneity within OC cell types.

## Cellular Diversity in Kolliker’s Organ

As discussed above, Kolliker’s organ is comprised of a population of epithelial cells located between the prosensory domain and the medial edge of the cochlear duct. The organ is transient as approximately 90% of the cells within the structure will undergo apoptosis prior to the onset of hearing leading to the formation of the inner sulcus which is lined by a monolayer of cuboidal epithelial cells, the last remnants of Kolliker’s organ (Knipper et al., 1999; Peeters et al., 2015). However, recent studies have demonstrated key roles for Kolliker’s organ in cochlear development. In particular, cells within the organ generate the bulk of the extracellular components that will form the tectorial membrane (Goodyear and Richardson, 2018) and spontaneous activity originating in Kolliker’s organ cells plays a key role in the organization of higher auditory centers (Tritsch et al., 2007; Tritsch and Bergles, 2010; Tritsch et al., 2010; Wang et al., 2015). Moreover, multiple studies have demonstrated that cells within Kolliker’s organ retain a high level of prosensory potential (Zheng and Gao, 2000; Woods et al., 2004; Ahmed et al., 2012; Kelly et al., 2012). Forced expression of *Atoh1* in Kolliker’s organ cells induces a HC-like phenotype and those *Atoh1* ^*+*^-cells then go on to induce surrounding cells to develop as SCs (Woods et al., 2004). As discussed, single cell analysis at E14 indicated a largely homogeneous population of Kolliker’s organ cells. But a similar analysis of the cochlear duct at E16 indicated four transcriptionally unique Kolliker’s organ clusters which increased to six by P1 (Figures 3D, 4A). Consistent with the decreasing size of Kolliker’s organ postnatally, only three clusters were present at P7 (Figure 4D). Interestingly, the different populations of Kolliker’s organ cells appear to be largely spatially separated along the medial-to-lateral axis of the cochlear duct. Labeling of Kolliker’s organ cells by *in situ* hybridization or immunolocalization indicates bands of expression of cluster-specific genes at specific locations along the medial-lateral border rather than diffuse expression throughout the organ (Okano et al., 2011; Okano and Kelley, 2013; Kolla et al., 2020). The roles of these different Kolliker’s organ cell types are just beginning to be understood.

One of the more intriguing aspects of Kolliker’s organ cells is the ability of some of these cells to develop as HCs or SCs under different circumstances. In addition to forced expression of Atoh1, inhibition of Sonic Hedgehog signaling leads to random patches of HCs and SCs within Kolliker’s organ (Driver et al., 2008). A key factor in the sensory potential of Kolliker’s organ cells appears to be expression of Sox2 and Jag1. At early time points, prior to E15, Sox2/Jag1 is expressed throughout much of Kolliker’s organ, and prosensory potential is also broad. As development continues, Sox2/Jag1 expression within Kolliker’s organ resolves to the two clusters located adjacent to the medial edge of the OC, as apparently, does prosensory potential. Consistent with this observation, a recent study that examined the sphere-forming potential of cells within the postnatal cochlea concluded that the bulk of the cells that can act as stem cells (as defined by forming spheres) are located in Kolliker’s organ, and, specifically, adjacent to the medial edge of the OC (Kubota et al., 2021). Finally, genetic lesioning of inner phalangeal cells during the early postnatal period induces Kolliker’s organ cells to move into the OC and to develop as replacement inner phalangeal cells, further demonstrating the developmental plasticity and prosensory potential in this cellular population (Mellado Lagarde et al., 2014). These results suggest an interesting evolutionary history for Kolliker’s organ. While soft tissues from transitional auditory organs have not been preserved, a reasonable inference is that there has been a progressive reduction in the size of the sensory epithelium over evolutionary time (Manley, 2012; Koppl and Manley, 2019). Does Kolliker’s organ represent a population of cells that were originally prosensory in nature and contributed to a larger mammalian sensory epithelium? If so, did those cells simultaneously provide the other functions noted above? Or were these new functions acquired after Kolliker’s organ cells lost their prosensory function? Inhibition of hedgehog signaling leads to random patches of sensory tissue in Kolliker’s organ. Is this signaling pathway one mechanism that was invoked to decrease the size of the sensory epithelium? Are there others and how would hearing be affected in a cochlea that contained significant HCs in the inner sulcus?

## New Regulators of the Development of the Lateral Prosensory Domain

Development of the lateral prosensory domain is regulated in large part through activation of Fgfr1 and then Fgfr3. But, deletion of either gene does not lead to an OC that is completely devoid of lateral cell types (Hayashi et al., 2007; Puligilla et al., 2007; Huh et al., 2012). In fact, in the case of Fgfr3, most lateral cell types, with the exception of inner pillar cells, are present. These results suggest that other signaling pathways must also play a role in development of this domain. One way to identify other potential candidates is through an analysis of differentially expressed genes in different single cell clusters. Analysis of the lateral prosensory domain cells at E14 identified *Transforming Growth Factor B Receptor 1* (*Tgfbr1*) and *Frizzled9* (*Fzd9*), as uniquely expressed in this population (Kolla et al., 2020) (Figure 3C). While a hearing phenotype has not been reported in viable *Fzd9* mutant mice (Ranheim et al., 2005), inhibition of Tgfbr1 *in vitro* significantly inhibited OHC formation, but had a minimal effect on IHCs (Kolla et al., 2020). Similarly, hearing loss has been reported in individuals with Loeys-Deitz syndrome, which can be caused by mutations in *Tgfbr1* (Van Laer et al., 2014; Riise et al., 2018; Takeda et al., 2018). Additional analysis of the genes and pathways that define medial and lateral prosensory clusters should provide new insights regarding the development of both domains and the unique cell types that are derived from each population.

## Specification of Hair Cell Phenotypes

One of the more intriguing applications of single cell approaches in developmental biology is the ability to treat the information from each cell as a single developmental data/time point. For instance, differentiation of the OC occurs in a gradient that begins in the basal region of the cochlea and extends, primarily, towards the apex. Therefore, a collection of single cells from a single time point contains cells at different stages of development. Ordering these cells from least mature to most mature creates a developmental trajectory of transcriptional expression. When this was done for OHCs collected at E14, E16, P1 and P7 in the Kolla et al. (2020) study, the results indicated four phases of unique transcriptional expression. While the trajectory was not studied in full, analysis of known genes largely confirmed the general accuracy of the analysis. *Sox2* and *Isl1* are only observed in phase 1 (Radde-Gallwitz et al., 2004; Deng et al., 2014), corresponding to E12-E14, while *Atoh1*, and *Pou4f3* show onset of expression in phase 2, corresponding to E14-E16 (Erkman et al., 1996; Xiang et al., 1998; Bermingham et al., 1999; Lanford et al., 2000; Driver et al., 2013) (Figure 4F). *Barhl1* and *Nhlh1* are upregulated in phase 3 (Li et al., 2002; Kruger et al., 2006), corresponding to E17-E19 and then *Tmc1* and, *Calb2* appear in stage 4 (Kurima et al., 2015), corresponding to postnatal time points, suggesting that this represents the final stage of functional onset (Figure 4F). An analysis of additional transcription factors using DAVID (Huang et al., 2007a; Huang et al., 2007b; Sherman et al., 2007) identified candidates, including *Bach2, Irx2*, *Zbtb8b, and Bhlhe40* (Chang and Kelley, unpublished), that await confirmation and analysis.

While the single cell RNAseq results described above have identified a number of candidate genes which may be involved in the development of OHCs, several recent papers have used other methods to identify genes that play key roles in OHC formation. First, screening for expression of the transcriptional inhibitor *Insm1* in the inner ear demonstrated that this gene is transiently expressed in OHCs between E15.5 and P2 (Figure 2B) (Lorenzen et al., 2015). In contrast, *Insm1* is never expressed in IHCs or vestibular HCs. Deletion of *Insm1* in the inner ear leads to disruption of expression of OHC-specific genes and an upregulation of expression of IHC-specific genes in some OHCs (Wiwatpanit et al., 2018). Moreover, overall cellular patterning in the OC is also disrupted and, consistent with these defects, significant elevations in ABR and DPOAE recordings were observed in *Insm1* conditional mutants. Population RNA sequencing confirmed that *Insm1* acts to repress a subset of early IHC-specific genes, suggesting that a first step in OHC differentiation may be to suppress an IHC-phenotype. Interestingly, some IHC-specific genes were not upregulated in *Insm1* mutant HCs, suggesting that other factors may also act to suppress the IHC phenotype. Moreover, many OHCs in *Insm1* conditional mutants expressed OHC-specific genes. Whether this represents limited recombination in the conditional mutants or roles for additional co-factors, such as *NeuroD2*, which is also expressed only in OHCs, remains to be determined.

A second study published in parallel with the Insm1 work used RiboTag to profile genes that are expressed in developing OHCs during the early postnatal and adult time period (Chessum et al., 2018). Bioinformatic analysis of the resulting datasets identified the transcription factor *Ikzf2* as strongly expressed in OHCs at all time points and immunolocalization confirmed OHC-specific expression of Ikzf2 beginning at P4. Inactivation of *Ikzf2* leads to OHC degeneration and hearing loss by P30, however the initial steps in OHC differentiation, including development of resting membrane potential and mechanotransduction currents are unaffected. Electromotility is also present in *Ikzf2* ^*−/−*^ OHCs although the magnitude of membrane shortening is reduced. A comparison of gene expression in OHCs from *Ikzf2* ^*−/−*^ and wildtype littermates indicated 36 genes that were down regulated and 105 genes that were upregulated in the absence of *Ikzf2* with several of the down-regulated genes already identified as OHC-specific. Finally, viral transfection was used to force expression of Ikzf2 in IHCs. Consistent with the mutant data, IHCs that express Ikzf2 show a partial transformation towards an OHC phenotype including a down-regulation of IHC-specific genes and an up-regulation of some OHC-specific genes and the development of stereociliary bundles with OHC-like morphologies. A subsequent study used a transgenic approach to confirm that forced expression of Ikzf2, in this case along with Atoh1, induces expression of *Slc26a5* (*Prestin*) and other OHC genes in SCs that have been induced to develop as HCs (Sun et al., 2021).

While the developmental processes that generate OHCs are not completely understood, the results described above are consistent with the following process. At the prosensory (precursor) stage, expression of set of unique transcription factors, which may include Prox1, induce a change in the competence of those prosensory cells, now referred to as lateral prosensory cells. As development continues, lateral prosensory cells are sorted into HC and SC fates, probably through a conserved Atoh1/Notch-mediated sorting process. Once a subset of the prosensory cells become committed to a HC fate, those cells activate an auditory HC differentiation program, that, if unaltered, would lead to the development of IHC-like cells. However, HCs derived from the lateral prosensory domain up-regulate expression of the repressor Insm1, along with possibly other repressors, which inhibits the IHC program. At the same time, additional transcription factors are probably required to drive these cells towards an OHC fate. While no factors have been shown to play a role in this process yet, scRNAseq results have identified some candidate genes. As development continues, other transcription factors, such as Ikzf2, are activated to drive specific aspects of OHC development. It is interesting to note that Ikzf2, while crucial for OHC development and function, does not appear to act as a master regulator for the late stages of OHC differentiation. Does this mean that another transcription factor might act up stream, activating Ikzf2 along with other factors that act in parallel? Alternatively, is Ikzf2 just one of a cascade of factors reflecting different steps in the evolution of the derived OHC phenotype? Additional experiments will clearly be required to discriminate between these possibilities.

## Conclusion

Over the course of the last 20 years, significant progress has been made in understanding the developmental processes and genetic signaling pathways that regulate development of the organ of Corti. However, many important questions remain unanswered. In particular, the signaling networks that are required to drive precursor cells to develop as mature OC cell types have not been discovered. For HCs, transcriptional networks are being assembled, but it is still not possible to drive a naïve cell to develop as a functional IHC or OHC. For SC types, even less is known. However, the advent of single cell technologies now allows us to characterize different cell types within the ear in terms of gene expression, epigenetic state and, potentially, protein expression. Moving forward, these techniques should lead to significant insights regarding the pathways that regulate the development of all the cell types with the organ of Corti.
